# Interplay between Bile Acids and Intestinal Microbiota: Regulatory Mechanisms and Therapeutic Potential for Infections

**DOI:** 10.3390/pathogens13080702

**Published:** 2024-08-20

**Authors:** Wenweiran Li, Hui Chen, Jianguo Tang

**Affiliations:** Department of Trauma-Emergency & Critical Care Medicine, Shanghai Fifth People’s Hospital, Fudan University, 128 Ruili Road, Shanghai 200240, China; lwwr1433248740@163.com

**Keywords:** bile acids, infection, microbiota

## Abstract

Bile acids (BAs) play a crucial role in the human body’s defense against infections caused by bacteria, fungi, and viruses. BAs counteract infections not only through interactions with intestinal bacteria exhibiting bile salt hydrolase (BSH) activity but they also directly combat infections. Building upon our research group’s previous discoveries highlighting the role of BAs in combating infections, we have initiated an in-depth investigation into the interactions between BAs and intestinal microbiota. Leveraging the existing literature, we offer a comprehensive analysis of the relationships between BAs and 16 key microbiota. This investigation encompasses bacteria (e.g., *Clostridioides difficile* (*C. difficile*), *Staphylococcus aureus* (*S. aureus*), *Escherichia coli*, *Enterococcus*, *Pseudomonas aeruginosa*, *Mycobacterium tuberculosis* (*M. tuberculosis*), *Bacteroides*, *Clostridium scindens* (*C. scindens*), *Streptococcus thermophilus*, *Clostridium butyricum* (*C. butyricum*), and lactic acid bacteria), fungi (e.g., *Candida albicans* (*C. albicans*) and *Saccharomyces boulardii*), and viruses (e.g., coronavirus SARS-CoV-2, influenza virus, and norovirus). Our research found that *Bacteroides*, *C. scindens*, *Streptococcus thermophilus*, *Saccharomyces boulardii*, *C. butyricum*, and lactic acid bacteria can regulate the metabolism and function of BSHs and 7α-dehydroxylase. BSHs and 7α-dehydroxylase play crucial roles in the conversion of primary bile acid (PBA) to secondary bile acid (SBA). It is important to note that PBAs generally promote infections, while SBAs often exhibit distinct anti-infection roles. In the antimicrobial action of BAs, SBAs demonstrate antagonistic properties against a wide range of microbiota, with the exception of norovirus. Given the intricate interplay between BAs and intestinal microbiota, and their regulatory effects on infections, we assert that BAs hold significant potential as a novel approach for preventing and treating microbial infections.

## 1. Introduction

The modulation of BAs in mammalian systems constitutes an intricate procedure, jointly orchestrated by the liver, intestines, and intestinal microbiota [1]. PBAs, predominantly comprising cholic acid and chenodeoxycholic acid (CDCA) [2], are largely reabsorbed within the enterohepatic circulation. These are subsequently converted by the intestinal microbiota, yielding SBAs, largely comprising lithocholic acid (LCA) and ursodeoxycholic acid (UDCA) [3,4]. In this metabolic cascading, enzymes engendered by the intestinal microbiota, particularly BSHs mediated by the bile salt hydrolase (BSH) gene and 7α hydroxylase facilitated by the *bai* operon, assume a pivotal role [5,6,7,8,9,10].

Both BAs and intestinal microbiota emerge as keystones in host metabolism, wherein their synthesized or regulated metabolites frequently function as signaling molecules, precluding the colonization of pathogens within the host [5,11].

The changes in the concentration and composition of intestinal BAs are not only pivotal in affecting the growth and colonization of various pathogens but also play a significant role in the mechanisms of disease prevention and pathogenesis [11,12]. Some studies have found that PBAs and SBAs play a vital role in maintaining intestinal homeostasis and combating infections [13,14,15]. For example, PBAs have been shown to facilitate the germination of *C. difficile* spores, while SBAs play a role in inhibiting its proliferation [16]. Interestingly, alterations in the intestinal microbiota significantly affect the host’s health and disease progression by profoundly influencing BAs’ conversion dynamics [17,18]. A wide array of intestinal microbiota exhibit BSH activity, which plays a crucial role in maintaining the balance of BA pools [9,10,19,20,21,22,23]. Moreover, the association between BSH activity and various health conditions, such as obesity, cancer, and inflammatory bowel disease, has become a burgeoning research hotspot [24,25,26,27,28,29,30], and BSHs are emerging as potential therapeutic targets for metabolic diseases [31,32,33,34,35].

Recent studies have highlighted the critical role of the *bai* operon-mediated 7α-dehydroxylation reaction in the intestinal microbiota, predominantly carried out by members of *Clostridium cluster* XIVa, particularly *Clostridium hiranonis* and *C. scindens* [36]. The *bai* operon consists of eight genes: seven encode enzymes and the eighth, *baiG*, encodes a transporter. This operon is conserved in every bacterial species known to 7α-dehydroxylate PBA, and its gene products are linked to specific steps in the pathway. The operon includes genes such as *baiB*, *baiCD*, *baiA2*, *baiE*, *baiF*, *baiH*, and *baiI*, each playing a unique role in the conversion of cholic acid to deoxycholic acid. The pathway involves both oxidative and reductive steps, with enzymes BaiB, BaiCD, BaiA2, BaiE, BaiF, and BaiH being necessary and sufficient for the complete conversion process [37]. This conversion not only increases the hydrophobicity of BAs but also triggers significant biological effects, including alterations in intestinal permeability, antibiotic biosynthesis, and activation of the Farnesoid X Receptor (FXR) [38,39,40]. The *bai* operon has shown effectiveness in reducing intestinal inflammation [41]. Furthermore, *C. scindens*, equipped with the *bai* operon, has demonstrated promise in combating *C. difficile* infections [42].

Among the diverse intestinal microbiota, *Firmicutes*, *Lactobacillus*, *Bifidobacterium*, *Enterococcus*, *Clostridium*, *Corbacteriaceae*, *Ruminococcaceae*, and *Clostridiaceae* exhibit BSH activity [36,43,44,45], while *Clostridium cluster* XIVa, particularly *Clostridium hiranonis* and *C. scindens*, as well as *Eubacterium* and *Peptostreptococcus*, possess 7α-dehydroxylase activity [36,37,46]. They are crucial for BA metabolism and maintaining intestinal homeostasis.

The dynamic interaction between BAs and the intestinal microbiota not only leads to changes in BA pools but also allows BAs to influence the structural composition of the intestinal microbiota [47]. Although earlier research focused on the interactions of PBAs or SBAs with specific intestinal microbiota, the transformation of PBAs to SBAs is an ongoing process facilitated by intestinal microbiota exhibiting BSH activity [48]. Increasing evidence suggests that the structure and function of the intestinal microbiota can exert long-lasting impacts on the host [49,50]. This review aims to offer a comprehensive exploration of the interactions between BAs and key intestinal microbes from the perspective of the intestinal microbiota. In the current era of widespread antibiotic use and rising microbial resistance [51], the role of BAs as preventive and therapeutic agents is becoming increasingly important.

## 2. Regulatory Mechanisms of BAs in Maintaining Intestinal Homeostasis and Counteracting Infections

BAs play a pivotal role in regulating intestinal homeostasis [52]. Some studies have shown that BAs can enhance intestinal epithelial permeability, thereby increasing susceptibility to infections [53]. Interestingly, natural BAs have demonstrated significant antimicrobial properties against a variety of organisms including bacteria, parasites, and fungi [54,55,56,57]. That is because the roles of PBAs and SBAs are different. For instance, PBAs like taurocholic acid (TCA) can promote *C. difficile* proliferation and facilitate *C. albicans* colonization [12,28,58,59,60]. In contrast, SBAs such as taurodeoxycholic acid can mitigate sepsis-induced intestinal inflammation, and deoxycholic acid and LCA encourage *C. scindens* proliferation and inhibit *C. difficile* spore germination [61,62,63,64]. These diverse effects could be attributed to specific BA species, the unique receptors they activate, and their interactions with intestinal microbiota.

**Figure 1:**
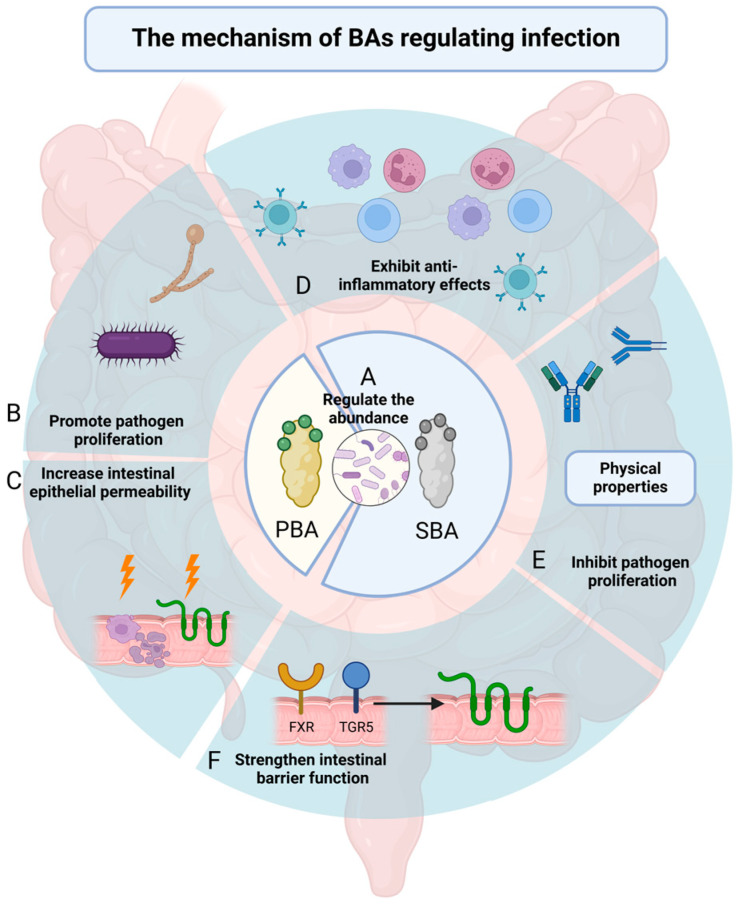
The mechanism of BAs regulating infection. BAs (**A**) regulate the abundance of intestinal microbiota. PBAs (**B**) promote pathogen proliferation and (**C**) increase intestinal epithelial permeability. SBAs (**D**) exhibit anti-inflammatory effects; (**E**) inhibit pathogen proliferation; and (**F**) strengthen intestinal barrier function. BA: bile acid; PBA: primary bile acid; SBA: secondary bile acid.

BAs interact with various cellular receptors, including FXR, TGR5 (Takeda G Protein-Coupled Receptor 5), Pregnane X Receptor, Sphingosine-1-Phosphate Receptor 2, and Vitamin D Receptor. FXR is activated primarily by CDCA. FXR activation strengthens the intestinal barrier, influences microbial community composition, and modulates inflammatory responses [65,66,67]. Moreover, FXR promotes the proliferation of regulatory T cells, enhancing their antiviral capabilities [68,69,70]. Taurodeoxycholic acid-induced TGR5 activation, which can reduce cAMP levels, inhibit the Myosin Light-Chain Kinase pathway and thus mitigate *Escherichia coli* epithelial barrier damage [71,72]. Other receptors such as Sphingosine-1-Phosphate Receptor 2, Pregnane X Receptor, and Vitamin D Receptor also play important roles in inflammatory response modulation when activated by BAs [73,74] (Figure 1).

### 2.1. BAs and Fungi

#### 2.1.1. Interactions between BAs and *Candida albicans*

*C. albicans*, an opportunistic fungus, primarily originates from its endogenous populations in the gastrointestinal tract [75,76,77,78,79,80,81]. *C. albicans* frequently causes invasive infections, particularly in immunocompromised individuals or in those with dysbiosis of the intestinal microbiota [58,78,82,83,84,85].

TCA, a primary bile acid, can modulate immune responses and microbial balance within the intestine, promoting the colonization and spread of fungi like *C. albicans* [86]. Specifically, TCA has been shown to suppress key immune molecules, such as angiogenin-4 and CX3CR1, which are crucial for maintaining intestinal barrier integrity [87,88,89]. Additionally, TCA is associated with reduced expression of tight junction proteins [90,91,92]. This may promote an increase in pathogen proliferation like *C. difficile* and facilitate *C. albicans* over-colonization [28,93,94,95,96]. In contrast, SBA, specifically LCA and DCA, can prevent *C. albicans* from transitioning from yeast to its virulent hyphal form and from its planktonic to biofilm phase, thereby restricting its proliferation in the intestine [96]. Additionally, SBAs can directly exhibit antimicrobial activity against *C. albicans* [96].

**Figure 2:**
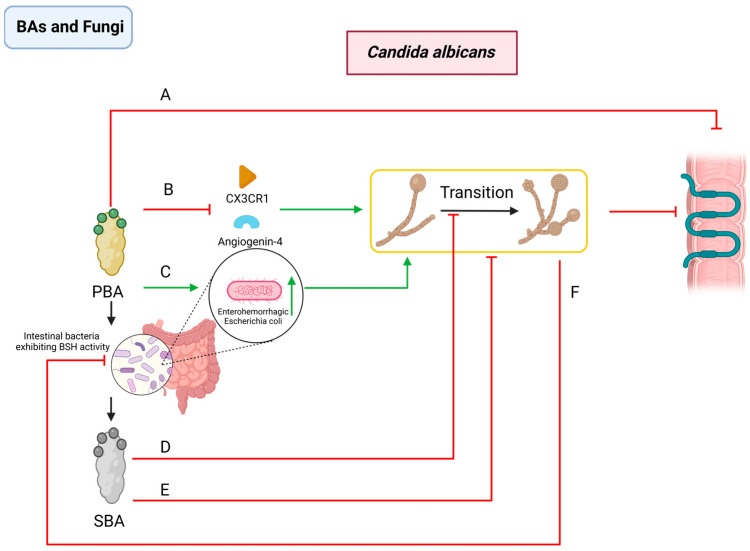
Interactions between BAs and *Candida albicans*. PBAs (**A**) reduce the tight junction proteins in the intestine; (**B**) inhibit the production of immune active substances angiogenin-4 and CX3CR1; (**C**) and increase the abundance of enterohemorrhagic *Escherichia coli*. SBAs (**D**) inhibit the transition of *C. albicans* from yeast to virulent hyphal form and from planktonic to biofilm phase and (**E**) direct antimicrobial activity against *C. albicans*. *C. albicans* (**F**) reduces the abundance of intestinal bacteria exhibiting BSH activity. PBA: primary bile acid; SBA: secondary bile acid; BSH: bile salt hydrolase; *C. albicans*: *Candida albicans*.

During mouse experiment investigations into intestinal microbiota composition following *C. albicans* infection, there was an increase in *Bacteroides*, *Proteobacteria*, *Pseudomonas*, and *Enterococcus* levels, while *Firmicutes* levels decreased [97,98]. These changes may facilitate enhanced *C. albicans* colonization by altering BSH activity and SBA concentrations in the intestine. Moreover, TCA supplementation can heighten *C. albicans*’s invasiveness and virulence by increasing specific bacterial populations, like enterohemorrhagic *Escherichia coli* [93] (Figure 2).

#### 2.1.2. Interactions between BAs and *Saccharomyces boulardii*

*Saccharomyces boulardii* CNCM I-745 (SB) has been shown to effectively mitigate the risk of *C. difficile* enteritis following antibiotic therapy in a clinical randomized controlled trial [99,100]. Central to the protective mechanism of SB is its ability to inhibit bacterial proliferation while rapidly restoring the balance of the intestinal microbiota [101]. In detail, SB can not only thwart bacterial adhesion but can also accelerate the neutralization of enteric toxins and bolster the immune response within the intestinal mucosa [102,103,104]. Furthermore, research involving healthy volunteer cohorts has illuminated that SB can safeguard the health of the intestine by promoting the proliferation of microbiota with BSH activity [28]. Complementing this, in vitro studies have also discovered that SB can hinder the germination of *C. difficile* spores [105,106,107].

### 2.2. BAs and Bacteria

#### 2.2.1. Interactions between BAs and *Clostridioides difficile*

*C. difficile* is a Gram-positive bacterium. *C. difficile* can produce two major protein toxins, TcdA and TcdB, which can disrupt host–cell signaling pathways and lead to apoptosis [108]. In clinical settings, *C. difficile* infections can range from mild diarrhea to severe pseudomembranous colitis [109].

**Figure 3:**
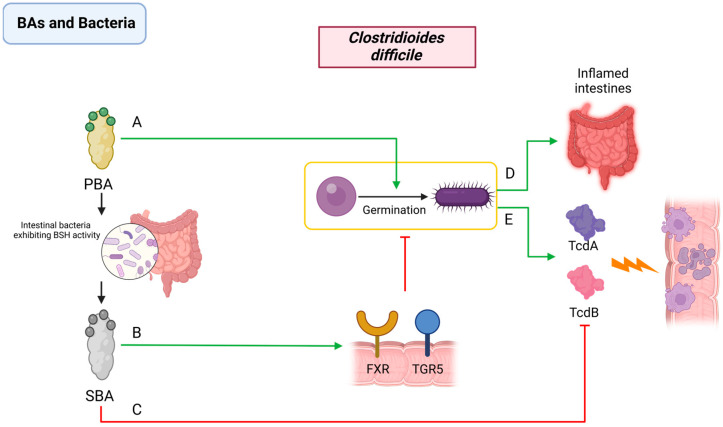
Interactions between BAs and *Clostridioides difficile*. PBAs (**A**) promote the spore germination of *C. difficile*. SBAs (**B**) bind to FXR and TGR5 receptors, activating NF-κB and other signaling pathways, enhancing innate immunity, and inhibiting the growth of *C. difficile*, and (**C**) interact with the C-terminus of toxin TcdB directly, inducing toxin structural changes, and preventing toxin binding with host cells. *C. difficile* (**D**) promotes the release of *C. difficile* toxins TcdA and TcdB and (**E**) induces intestinal inflammation. PBA: primary bile acid; SBA: secondary bile acid; FXR: Farnesoid X Receptor; TGR5: Takeda G Protein-Coupled Receptor 5; *C. difficile*: *Clostridioides difficile*.

In the lifecycle of *C. difficile*, BAs play a regulatory role [110,111]. Some studies have identified that BAs can affect the proliferation of *C. difficile* by influencing both the structural and functional aspects of the TcdB toxin [97,112]. In addition, *C. difficile* spores can detect specific BAs as environmental cues in the gastrointestinal tract and initiate germination processes [109,113,114]. Specifically, TCA, a primary bile acid, has been implicated in facilitating the in vitro germination of *C. difficile* spores, which can promote the subsequent release of toxins [115]. Conversely, SBAs like LCA and deoxycholic acid are known to inhibit the growth and toxic effects of *C. difficile* [110,116,117]. This inhibition includes (1) the activation of BA receptors such as FXR and TGR5 by SBAs, which enhances the innate immune response and inhibits *C. difficile* proliferation through signaling pathways, notably NF-κB [118], and (2) the direct interaction of SBAs with the C-terminal region of TcdB, leading to conformational changes in the toxin and preventing its binding and toxic effects on host cells [119] (Figure 3).

#### 2.2.2. Interactions between BAs and *Staphylococcus aureus*

*S. aureus*, a Gram-positive bacterium, presents significant clinical management challenges, which are exacerbated by indiscriminate antibiotic use [120]. Recent studies, though limited in number, with only two studies identified so far, have begun to elucidate the significant role of SBAs in the response to *S. aureus* infections.

Deoxycholic acid, a secondary bile acid, has been observed to promote the repair of tight junction proteins in the blood–milk barrier and substantially reduce the expression of inflammation-associated markers in mouse experiments involving *S. aureus*-induced mastitis [8,120]. Furthermore, deoxycholic acids can also alleviate *S. aureus*-induced endometritis discovered in Hu J’s studies [121]. Their protective effects are thought to stem from deoxycholic acid’s influence on the TGR5/PKA-NF-κB-NLRP3 inflammasome signaling axis [122]. However, deoxycholic acid does not directly suppress the proliferation of *S. aureus* [8].

Additionally, studies indicate that an imbalance in intestinal microbiota leads to an exacerbated response to mastitis in mouse experiments challenged with *S. aureus*, thereby intensifying the clinical symptoms [123,124]. Remarkably, supplementing the intestinal microbiota of infected mice with BSH-active organisms, such as *C. scindens*, significantly reduces the inflammatory response to mastitis [8].

#### 2.2.3. Interactions between BAs and *Enterococci*

In the gastrointestinal tract, *Enterococcus faecalis* (*E. faecalis*) is a commensal bacterium. However, under conditions of intestinal microbiota dysbiosis, *E. faecalis* may transition to a pathogenic state, particularly in elderly or immunocompromised individuals [125,126,127]. Recent clinical studies have elucidated that the elevation of deoxycholic acid levels or a reduction in TCA levels can effectively curtail the proliferation of *E. faecalis*. Further research suggests that deoxycholic acid’s growth-inhibitory effect on *E. faecalis* could be due to its impact on the expression of various ribosomal protein genes [128].

**Figure 4:**
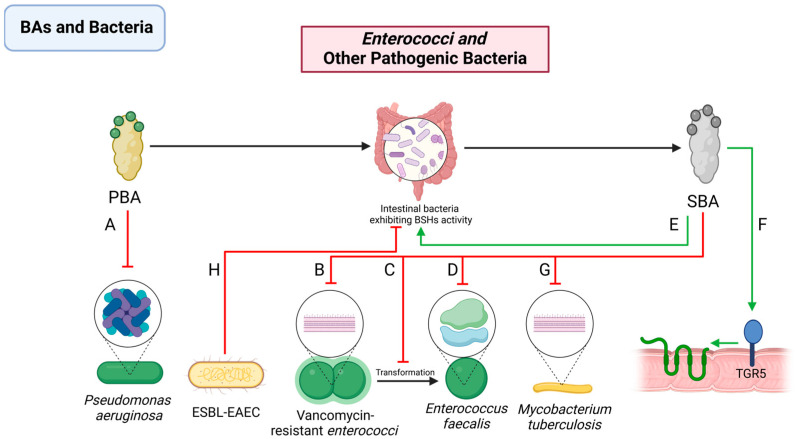
Interactions between BAs and *Enterococci* and other pathogenic bacteria. PBAs (**A**) regulate the synthesis of virulence-related metabolites, such as the iron chelator pyochelin, thereby affecting *Pseudomonas aeruginosa*’s toxicity, and inhibit its biofilm formation. SBAs (**B**) inhibit the expression of ribosomal protein genes, suppressing the growth of *E. faecalis*; (**C**) maintain VRE in a diplococcal state and inhibit the morphological transformation of VRE; (**D**) inhibit the formation of *VRE* biofilms; (**E**) optimize the structure of the intestinal microbiota; (**F**) increase TGR5 transcription, thereby enhancing innate immunity, and strengthen the intestinal barrier; and (**G**) disrupt the cell wall of lipid-rich *M. tuberculosis*. ESBL-EAEC (**H**) reduces the abundance of intestinal bacteria exhibiting BSH activity. PBA: primary bile acid; SBA: secondary bile acid; VRE: *vancomycin-resistant enterococci.* TGR5: Takeda G Protein-Coupled Receptor 5.

Vancomycin-resistant *enterococci* (VRE) present significant challenges in clinical settings due to their antibiotic resistance. The formation of biofilms is critical for the colonization of enterococci in various host environments [129]. Rahman’s study has revealed that LCA can curtail the growth of VRE by maintaining VRE in a diplococcal state and inhibiting the morphological transformation of VRE. Additionally, LCA exposure induces genetic mutations in VRE that result in persistent diplococcal morphology and reduced biofilm production [130] (Figure 4B–D).

#### 2.2.4. Interactions between BAs and Other Bacteria (Extended-Spectrum Beta-Lactamase-Resistant *Escherichia coli*, *Mycobacterium tuberculosis*, *Pseudomonas aeruginosa*, etc.)

The overuse of antibiotics has led to a widespread increase in the prevalence of extended-spectrum beta-lactamase-resistant *Escherichia coli* (ESBL-EAEC). The pathological hallmarks of ESBL-EAEC infection include inflammation, epithelial cell exfoliation, and compromised epithelial barrier functionality [131]. UDCA, a secondary bile acid, has shown significant inhibitory effects on ESBL-EAEC in mouse experiments. In the context of ESBL-EAEC infection, a notable reduction in the abundance of key intestinal microbial families with BSH activity such as *Corbacteriaceae*, *Ruminococcaceae*, and *Lachnospiraceae* has been observed. However, this change is effectively countered by UDCA treatment by repairing microbial imbalances [44]. Moreover, UDCA enhances tight junction functionality by upregulating TGR5 transcription and inhibiting IκB α phosphorylation [14,132] (Figure 4E,F,H).

*M. tuberculosis*, the causative agent of tuberculosis, shows a unique susceptibility pattern in the gastrointestinal tract [133]. Regions with lower BA concentrations, such as the terminal ileum and cecum, are more susceptible to intestinal tuberculosis [134]. BAs like CDCA, deoxycholic acid, and cholic acid have demonstrated inhibitory effects on the proliferation of *M. tuberculosis*. This inhibition could be due to the detrimental impact of BAs on the distinctive lipid-rich cell wall of *M. tuberculosis* [135] (Figure 4G).

*Pseudomonas aeruginosa* is known for its diverse infection profiles [136]. Surprisingly, TCA, as a primary bile acid, demonstrates a significant inhibitory effect on *Pseudomonas aeruginosa*. In detail, TCA is particularly effective in inhibiting biofilm formation and dispersing existing biofilms [137,138]. This effect is believed to originate from TCA’s modulation of *Pseudomonas aeruginosa*’s virulence factors, including its impact on metabolites like the siderophore pyochelin, thereby altering its toxicity and biofilm dynamics [139] (Figure 4A).

Moreover, BAs influence various other pathogenic bacteria. For example, deoxycholic acid has been shown to induce the transcription of genes involved in DNA repair and recombination in response to infections by bacteria such as *Escherichia coli*, *Salmonella enterica* serovar Typhimurium, *Bacillus cereus*, and *Listeria monocytogenes* [140]. However, BAs also have a dual role; their presence has been linked to increased virulence in *Shigella dysenteriae*, promoting infection [141].

#### 2.2.5. Interactions between BAs and *Bacteroidetes*

The Bacteroidetes phylum significantly contributes to gastrointestinal health and the prevention of infections [142]. It has been reported that *Bacteroides thetaiotaomicron* (*B. thetaiotaomicron*), *Bacteroides ovatus,* and *Bacteroides fragilis* can alleviate colitis in mouse experiments by promoting the production of SBAs to inhibit the proliferation of *C. difficile* [143,144,145,146].

In related research, the *Bacteroides dorei* strain (BDX-01) and its therapeutic effects were investigated in a colitis mouse model by regulating BA metabolism, indicated by changes in total fecal BA levels and BA ratios, and by affecting the FXR-NLRP3 inflammasome signaling pathway, which led to reduced proinflammatory cytokine expression and diminished IL-1β secretion in the colon, thereby mitigating DSS-induced experimental colitis [9,147,148,149,150,151].

**Figure 5:**
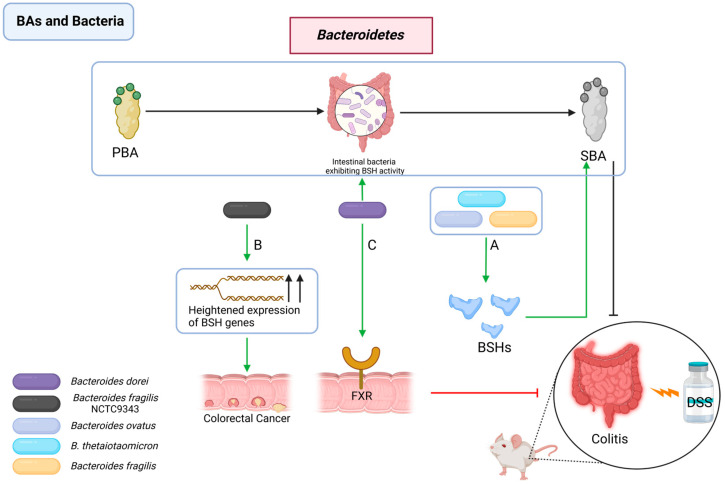
Interactions between BAs and *Bacteroidetes*. (**A**) *Bacteroidetes* exhibit BSH activity, facilitate SBA production, and alleviate colitis; (**B**) *Bacteroides* with high BSH gene expression will promote the massive production of SBA, which can induce colorectal cancer; (**C**) BDX-01 enhances intestinal health by modulating BA metabolism and the FXR-NLRP3 inflammasome signaling pathway, thus mitigating experimental colitis. PBA: primary bile acid; SBA: secondary bile acid; BSH: bile salt hydrolase; FXR: Farnesoid X Receptor.

However, a potential adverse role of *Bacteroides fragilis* NCTC9343 in gastrointestinal health has been revealed, particularly concerning their BSH activity [152]. Elevated BSH gene expression in colonizing Bacteroidetes strains can lead to an increased influx of BAs, which may activate signaling pathways like WNT/β-catenin and NF-κB, resulting in oxidative DNA damage and enhanced cellular proliferation, eventually exacerbating colorectal cancer progression in mouse experiments [9,34,153] (Figure 5).

#### 2.2.6. Interactions between BAs and *Clostridium scindens*

**Figure 6:**
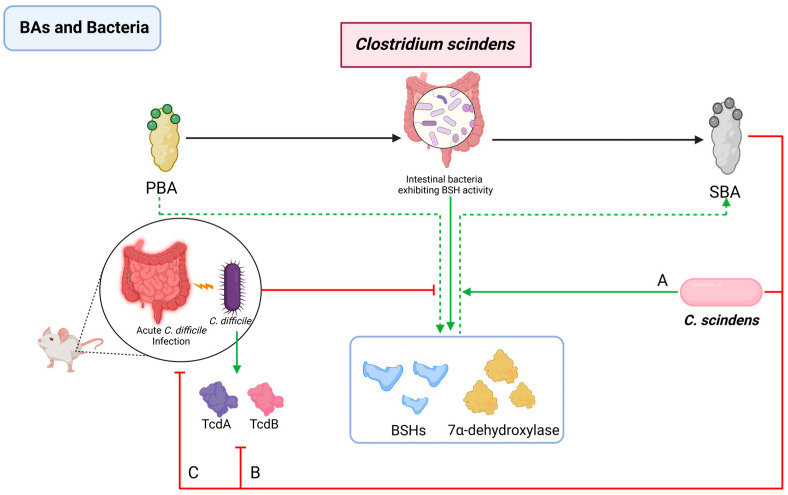
Interactions between BAs and *Clostridium scindens*. *C. scindens* (**A**) exhibits BSH and 7α-dehydroxylase activity, facilitates SBA production, and inhibits *C. difficile* infection; (**B**) inhibitd the toxin production of *C. difficile*; and (**C**) reduces *C. difficile* overall count. PBA: primary bile acid; SBA: secondary bile acid; BSH: bile salt hydrolase; *C. difficile*: *Clostridioides difficile*; *C. scindens*: *Clostridium scindens*.

*C. scindens* harbors a bile acid-inducible operon, the *bai* operon [61]. This operon is essential for the synthesis of SBAs by regulating the expression of 7α-dehydroxylase [7,62]. Some studies have discovered that C. scindens plays a crucial role in preventing the colonization and proliferation of C. difficile [41]. In cases of acute C. difficile infection, a marked decrease in both BSH and 7α-dehydroxylase expression is observed in the cecal contents of mice, aligning with reduced gene expressions in the Lachnospiraceae and Clostridiaceae families [154]. However, introducing C. scindens into the gut of mice with acute C. difficile infection significantly enhances intestinal health. Particularly, C. scindens has been shown to suppress TcdA/TcdB toxin production by C. difficile and reduce its overall count by inhibiting biofilm formation [41,110,155,156]. Therefore, the synergistic action of SBAs and C. scindens is increasingly recognized as a critical strategy in countering intestinal colonization by this pathogenic bacterium [157] (Figure 6).

#### 2.2.7. Interactions between BAs and *Clostridium butyricum*

*C. butyricum* can modulate lipid metabolism by influencing the BA profile within the liver and ileum [158,159]. Research has shown that *C. butyricum* supplementation can reshape the intestinal microbiota composition and BA distribution of intrauterine growth-restricted piglets, thereby optimizing their lipid metabolism. At the same time, it significantly reduces the abundance of specific intestinal microbiota *Streptococcus* and *Enterococcus* in the ileum of these piglets, leading to an increase in conjugated bile acid (CBA). This increase in CBA, which can be derived from both PBAs and SBAs through conjugation with amino acids like glycine or taurine, activates key liver receptors, such as liver X receptor α (LXRα) and FXR, which are crucial for reducing inflammatory responses and protecting normal liver function [107,160,161,162,163,164,165,166,167].

**Figure 7:**
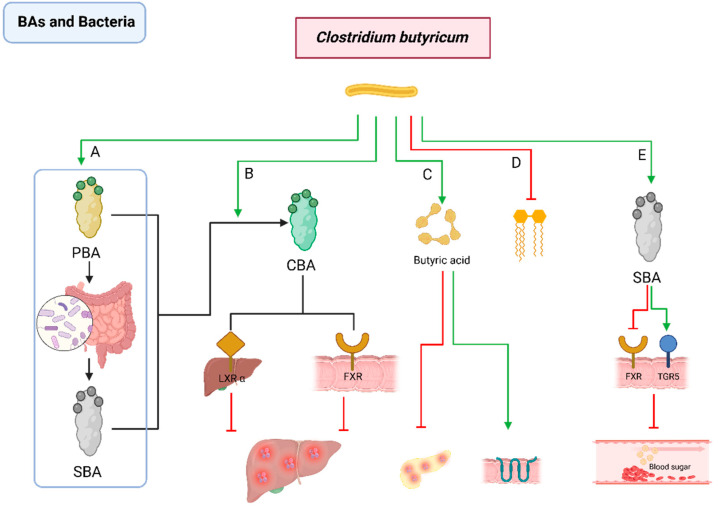
Interactions between BAs and *Clostridium butyricum*. *C. butyricum* (**A**) modulates the ratio of PBAs to SBAs and (**B**) promotes the production of CBA. CBA improves intrauterine growth restriction and reduce liver inflammation by activating LXRα and FXR; (**C**) enhances the production of butyric acid, which ameliorates chronic pancreatitis and strengthens the tight junctions of intestinal epithelial cells, thereby reducing intestinal barrier damage and restoring the intestinal immune microenvironment; (**D**) inhibits lipid synthesis; and (**E**) coordinates SBA regulation to activate FXR and inhibit TGR5, thereby regulating blood sugar and reducing obesity. PBA: primary bile acid; SBA: secondary bile acid; BSH: bile salt hydrolase; LXRα: liver X receptor alpha; FXR: Farnesoid X Receptor; CBA: conjugated bile acid; *C. butyricum*: *Clostridium butyricum*.

Specially, *C. butyricum* strain CCFM1299 administration leads to a significant increase in UDCA levels in feces and taurocholic acid levels in serum, thereby activating TGR5 and inhibiting FXR, subsequently enhancing GLP-1 production in the intestine, which helps regulate blood sugar and reduce obesity. This effect has been observed in experiments using a high-fat diet mouse model [168,169,170,171]. Furthermore, *C. butyricum* reshapes the microbiota by increasing butyric acid levels, maintaining SBA balance, and attenuating the inhibitory effects of the FXR/SHP pathway on lipid synthesis [172]. And it also activates the butyrate/GPR43 pathway, reducing damage to the intestinal barrier and restoring the intestinal immune microenvironment in rabbits with chronic pancreatitis [173] (Figure 7).

#### 2.2.8. Interactions between BAs and Lactic Acid Bacteria

*Pediococcus pentosaceus* Li05 belong to the *Pediococcus* genus of the *Lactobacillaceae* family. Li05 can improve tight junction proteins and downregulate inflammatory responses in mouse experiments by modulating intestinal microbiota and BA metabolism [174]. Specifically, in an acute C. difficile infection mouse model, it has been shown to promote the growth of beneficial microbial taxa such as Lactobacillus, Prevotella, and Paraprevotella while inhibiting opportunistic pathogens. This modulation of the intestinal microbiota leads to alterations in BA composition, which subsequently influences liver injury processes [59,175]. Additionally, it has been reported that Li05 treatment notably reduced weight loss, liver damage, and bile stasis in 3,5-Diethoxycarbonyl-1,4-Dihydrocollidine-induced cholestasis mouse experiments [176,177], which is likely linked to Li05’s modulation of the intestinal microbiota, particularly enhancing propionate- and butyrate-producing bacteria like Anaerostipes and Eubacterium. Anaerostipes and Eubacterium are known for metabolizing inositol into propionic and butyric acids and converting PBAs into SBAs via 7α-dehydroxylation [19,178,179].

Liu L et al. also revealed that Lactiplantibacillus plantarum LPJZ-658 modulates intestinal microbiota and BA metabolism in mouse models, which reveals the potential for treating non-alcoholic fatty liver disease [180]. Furthermore, Lactiplantibacillus plantarum LPJZ-658 increased the abundance of Firmicutes and Actinobacteria, suggesting a healthier intestinal environment conducive to non-alcoholic steatohepatitis mitigation [181,182,183].

#### 2.2.9. Interactions between BAs and *Streptococcus thermophilus*

*Streptococcus thermophilus* MN002 *(S. thermophilus*), acknowledged as an efficacious probiotic [184,185], has shown promising potential in mitigating the risks associated with metabolic syndrome and colorectal tumors [186,187,188], as well as reducing the incidence of obesity, neonatal bacteremia, and meningitis caused by *Escherichia coli* K1 [189]. The consumption of a high-fat diet is known to disrupt the intestinal microbial equilibrium, leading to both intestinal and systemic inflammation [190,191,192]. Intriguingly, deoxycholic acid can reduce the inflammatory symptoms in high-fat diet mouse experiments. Specifically, *S. thermophilus* is capable of optimizing BA configurations and fostering a balanced intestinal microbiota [193,194]. This is achieved by augmenting the relative abundance of bacteria proficient in producing SBAs, including members of the *Ruminococcaceae*, *Bacteroides*, *Clostridium*, and *Blautia* families [45].

### 2.3. BAs and Viruses

#### 2.3.1. Interactions between BAs and Coronavirus SARS-CoV-2

Severe Acute Respiratory Syndrome Coronavirus 2 (SARS-CoV-2) utilizes the receptor-binding domain within its spike protein to engage the host’s angiotensin-converting enzyme 2 (ACE2) receptor, facilitating cellular entry [195,196,197]. Recent investigations have revealed the potential of BAs, particularly UDCA and CDCA, in disrupting this critical virus–host interaction.

Some studies have identified that UDCA can directly bind the receptor-binding domain of SARS-CoV-2, thereby diminishing its affinity for ACE2 and potentially mitigating cellular damage [198,199,200]. Specifically, UDCA appears to alter the virus’s structural integrity, allowing the penetration of polar inhibitors and solvents into the viral cells, which could impede replication [200,201].

Beyond direct antiviral effects, UDCA can also modulate the host’s immune response. The cytokine storm, a critical factor in severe COVID-19 cases, can be mitigated by UDCA’s anti-inflammatory, antioxidant, immunomodulatory, and anti-apoptotic properties [202,203,204,205,206,207]. Notably, UDCA can also reduce FXR expression in various human and animal tissues by regulating ACE2 transcription [208,209,210,211,212]. In addition, retrospective studies have indicated that UDCA can improve clinical outcomes in patients [213]. However, UDCA did not demonstrate a reduction in susceptibility to SARS-CoV-2 infection in pediatric populations [214].

**Figure 8:**
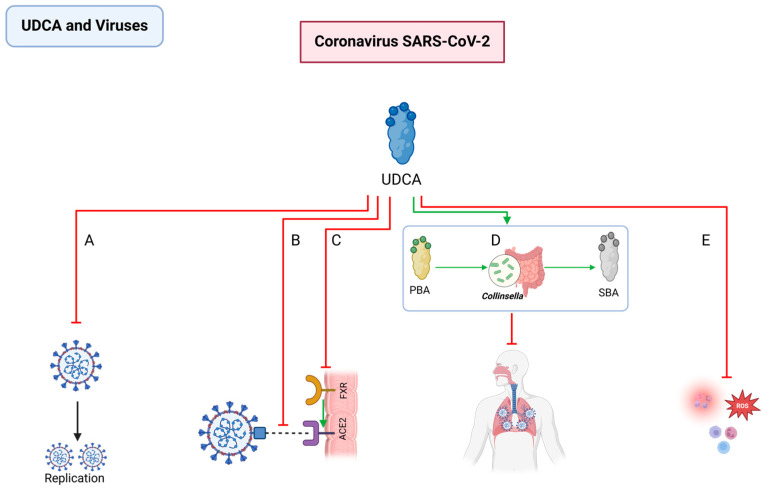
Interactions between UDCA and Coronavirus SARS-CoV-2. UDCA (**A**) directly damages the virus structure, inhibiting its replication; (**B**) reduces the affinity between the receptor-binding domain of coronavirus SARS-CoV-2 and the host ACE2; (**C**) inhibits FXR gene expression, thereby suppressing ACE2 expression; (**D**) increases the abundance of *Collinsella* and promotes the synthesis of 7β-Hydroxysteroid dehydrogenase, ameliorating acute respiratory distress syndrome in COVID-19; and (**E**) possesses anti-inflammatory, antioxidative, immunomodulatory, and anti-apoptotic properties. UDCA: ursodeoxycholic acid; SARS-CoV-2: Severe Acute Respiratory Syndrome Coronavirus 2; FXR: Farnesoid X Receptor; ACE2: angiotensin-converting enzyme 2; SBA: secondary bile acid.

Emerging research suggests a correlation between the intestinal microbiome, particularly the *Collinsella* genus, and COVID-19 outcomes. Hirayama M et al. employed machine learning to uncover a potential link between intestinal *Collinsella* and reduced COVID-19 severity [215]. UDCA produced by *Collinsella* may prevent COVID-19 infection and ameliorate acute respiratory distress syndrome in COVID-19 by suppressing cytokine storm syndrome in clinical setting [216] (Figure 8).

#### 2.3.2. Interactions between BAs and Other Viruses (Influenza Virus, Norovirus, etc.)

Influenza A virus (IAV) is a significant respiratory pathogen. Recent studies have uncovered the antiviral potential of CDCA and sodium taurocholate against IAV. They attenuate IAV infection by inhibiting the nuclear export of viral ribonucleoproteins and modulating the Toll-like receptor 4/NF-κB signaling pathway [217,218]. Specifically, CDCA, a secondary bile acid, shows promise in inhibiting IAV subtypes, including H5N1, H9N2, and H1N1, by interfering with viral ribonucleoproteins’ nuclear export and inhibiting viral replication [217]. Sodium taurocholate, a primary bile acid derivative, surprisingly exhibits antiviral efficacy against various influenza strains, including H5N6 and H3N2, by targeting the early stages of viral transcription and replication via the TLR4/NF-κB pathway [219].

BAs play a interesting role in norovirus infection [220,221]. Glycine deoxycholic acid, a secondary bile acid, enhances murine noroviruses’ infectivity [222]. In addition, the intestinal microbiota distinctly modulates norovirus infection dynamics in different intestinal regions, with BAs mediating their inhibitory effect in the proximal small intestine, while BA receptors regulate infection in the distal small intestine [223,224].

**Figure 9:**
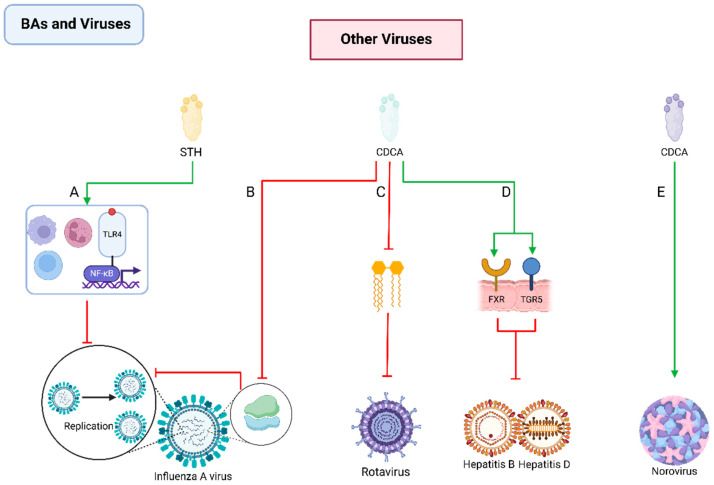
Interactions between BAs and Other Viruses. (**A**) STH manifests antiviral activity against IVA infections through the modulation of signaling pathways, including TLR4/NF-κB; (**B**) CDCA demonstrates the capacity to attenuate IAV infections by inhibiting the nuclear export of vRNPs; (**C**) CDCA can reduce virus-induced lipid synthesis, inhibiting the replication of rotavirus; (**D**) CDCA activates FXR and TGR5 receptors to counteract HBV infection; (**E**) GCDCA enhances the virulence of norovirus through a mechanism that is not yet clarified. STH: sodium taurocholate; TLR4: Toll-like receptor 4; CDCA: chenodeoxycholic acid; vRNPs: viral ribonucleoproteins; IAV: influenza A virus; GCDCA: glycine deoxycholic acid; FXR: Farnesoid X Receptor; TGR5: Takeda G Protein-Coupled Receptor 5; HBV: hepatitis B virus.

Moreover, CDCA has shown inhibitory effects against digestive system viruses, including rotavirus, hepatitis B, and hepatitis D viruses [68,225]. Specifically, CDCA activates FXR and TGR5 receptors in HBV infections in mouse experiments. Also, CDCA can inhibit the replication of rotavirus by reducing virus-induced lipid synthesis [69,218] (Figure 9).

## 3. Conclusions

The regulation of BAs is a complex process in mammalian systems. Intestinal microbiota play a crucial role in converting PBAs to SBAs by regulating the metabolic activities of BSHs and 7α-hydroxylase. Among the diverse intestinal microbiota, *Firmicutes*, *Lactobacillus*, *Bifidobacterium*, *Enterococcus*, *Clostridium*, *Corbacteriaceae*, *Ruminococcaceae*, and *Clostridiaceae* exhibit BSH activity [36,43,44,45] while *Clostridium cluster* XIVa, particularly *Clostridium hiranonis* and *C. scindens*, as well as *Eubacterium* and *Peptostreptococcus*, possess 7α-dehydroxylase activity [36,37,46]. They are crucial for BA metabolism and maintaining intestinal homeostasis.

Here, we explored the interactions between BAs and a comprehensive array of 16 key intestinal microbiota. Our research found that *Bacteroides*, *C. scindens*, *Streptococcus thermophilus*, *Saccharomyces boulardii*, *C. butyricum*, and lactic acid bacteria can regulate the metabolism and function of BSHs and 7α-dehydroxylase. BSHs and 7α-dehydroxylase play crucial roles in the conversion of PBAs to SBAs. It is important to note that PBAs generally promote infections, while SBAs often exhibit distinct anti-infection roles. In the antimicrobial action of BAs, SBAs demonstrate antagonistic properties against a wide range of microbiota, with the exception of norovirus.

SBAs combat infections in several ways. First, SBAs slow down the growth of pathogen proliferation, inhibit the transformation of *C. albicans*, reduce *C. difficile* spore sprouting, disrupt VRE biofilms, and weaken *M. tuberculosis* cell walls. SBAs also reduce SARS-CoV-2’s binding to ACE2 receptors and inhibit influenza virus replication. Second, SBAs modify the structure of *C. difficile*’s TcdB toxin and trigger the NF-κB signaling pathway via BA receptors like FXR and TGR5. This interaction boosts the body’s immune defenses, enhancing responses against pathogens like *C. difficile* and SARS-CoV-2. Last, the synergy between SBAs and some specific intestinal microbiota is crucial, particularly in enhancing their anti-infective potential. *C. butyricum*, for example, promotes intestinal health through enterohepatic circulation, reducing BSH-active microbiota and increasing CBA production. However, certain *Bacteroidetes* strains with high BSH gene expression may inadvertently increase BA entry into the colon, potentially triggering colorectal cancer.

The interaction between viruses and BAs is complex. Most SBAs preserve intestinal mucosal health, but glycine deoxycholic acid, a secondary bile acid, potentially exacerbates norovirus infection. In addition, STH is a primary bile acid derivative and surprisingly shows efficacy against the influenza virus.

BAs are diverse, each possessing unique physical structures and biological properties. The dynamic metabolism of BAs in the human body results in fluctuations in their types and concentrations along the intestinal tract. Current research, often utilizing fixed BA formulations, may not fully capture these variations. Additionally, it is important to note that most interactions between BAs and microbiota have been studied in vitro. However, the in vivo effects may differ significantly due to the complex intestinal environment. For example, BAs can alter the mucus layer, which in turn affects pathogenicity, the effectiveness of antimicrobials, and immune responses. These in vivo dynamics remain largely unstudied, indicating the need for further research to fully understand BAs’ therapeutic potential. Nonetheless, it is evident that SBAs generally exert a favorable anti-infectious influence against most microbiota-induced infections.

Given the intricate interplay between BAs and intestinal microbiota, and their regulatory effects on infections, we assert that BAs hold significant potential as a novel approach for preventing and treating microbial infections.

## Abbreviations

BAs	Bile acids
BSHs	Bile salt hydrolases
BSH	Bile salt hydrolase
CBA	Conjugated bile acid
PBA	Primary bile acid
CDCA	Chenodeoxycholic acid
TCA	Taurocholic acid
SBA	Secondary bile acid
LCA	Lithocholic acid
UDCA	Ursodeoxycholic acid
FXR	Farnesoid X Receptor
LXRα	Liver X receptor α
TGR5	Takeda G Protein-Coupled Receptor 5
ACE2	Angiotensin-converting enzyme 2
*C. albicans*	*Candida albicans*
*C. difficile*	*Clostridioides difficile*
*C. scindens*	*Clostridium scindens*
*E. coli*	*Escherichia coli*
*S. aureus*	*Staphylococcus aureus*
*E. faecalis*	*Enterococcus faecalis*
*ESBL-EAEC*	Extended-spectrum beta-lactamase-resistant *Escherichia coli*
*VRE*	*Vancomycin-resistant enterococci*
*M. tuberculosis*	*Mycobacterium tuberculosis*
*S. thermophilus*	*Streptococcus thermophilus*
*SB*	*Saccharomyces boulardii*
*C. butyricum*	*Clostridium butyricum*
SARS-CoV-2	Severe Acute Respiratory Syndrome Coronavirus 2
IAV	Influenza A virus
COVID-19	Coronavirus Disease 2019
PBA Examples	Cholic acid, chenodeoxycholic acid, taurocholic acid, and sodium taurocholate
SBA Examples	Lithocholic acid, deoxycholic acid, ursodeoxycholic acid, taurodeoxycholic acid, and glycine deoxycholic acid
